# Supplementary material for: Robot pouring: identifying causes of spillage and selecting alternative action parameters using probabilistic actual causation
Source: Front Cognit. 2025 Jun 20;4:1565059. doi: 10.3389/fcogn.2025.1565059 (PMC13281113; doi:10.3389/fcogn.2025.1565059)
Supplement: Supplementary file 1 [file Data_Sheet_1.PDF]

Supplementary Material

1 CAUSAL DISCOVERY USING THE PC ALGORITHM

1.1 Tetrad setup and bootstrapping results

Figure S1 shows the exact parameters used in Tetrad to run the PC algorithm. The specification of background knowledge in the form of tiers is shown in Figure S2. Note that within-tier edges are allowed in the background knowledge.

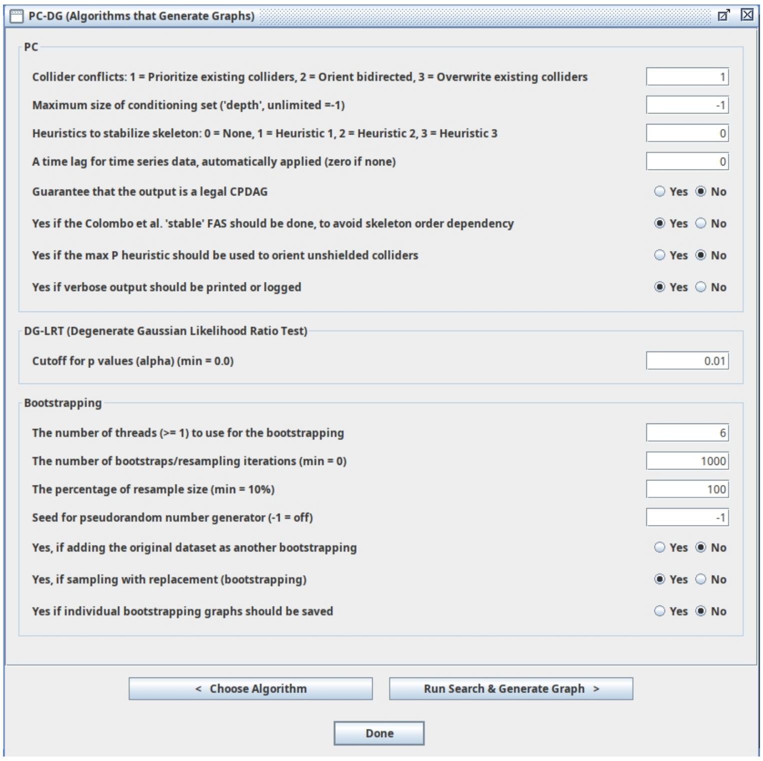

Figure S1. PC and DG parameters specified in Tetrad.

To validate the causal relationships inferred from the data, we performed a bootstrapping analysis. Conducting a bootstrapping analysis is recommended for causal discovery(Malinsky and Danks, 2017; Glymour et al., 2019). We ran the PC algorithm on 1000 bootstraps, producing 1000 different structures. The edge-type frequencies obtained from bootstrapping indicate whether the discovered causal relationships are stable across different bootstrap samples (Glymour et al., 2019). In Figure S3, we report the frequency of the edge types between variables. For ease of interpretation and comparison, the frequency of edge type is reported as a proportion of the number of bootstraps. Given the different edge types (including "no edge"), we interpret an edge frequency larger than 0.5 as stable. The reported DAG, termed *discovered DAG* includes the stable edges and no-edges. The PC algorithm discovered direct edges, edges that are definitely direct (DD), and undirected edges (i.e., the data are consistent with $X \to Y$ and $X \leftarrow Y$ (Malinsky and Danks, 2017)).

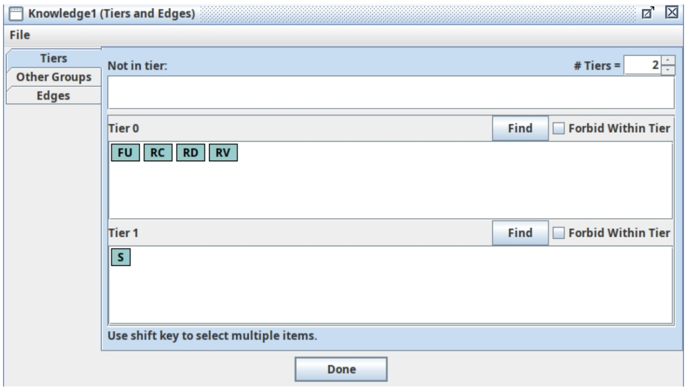

Figure S2. Background knowledge specified in Tetrad.

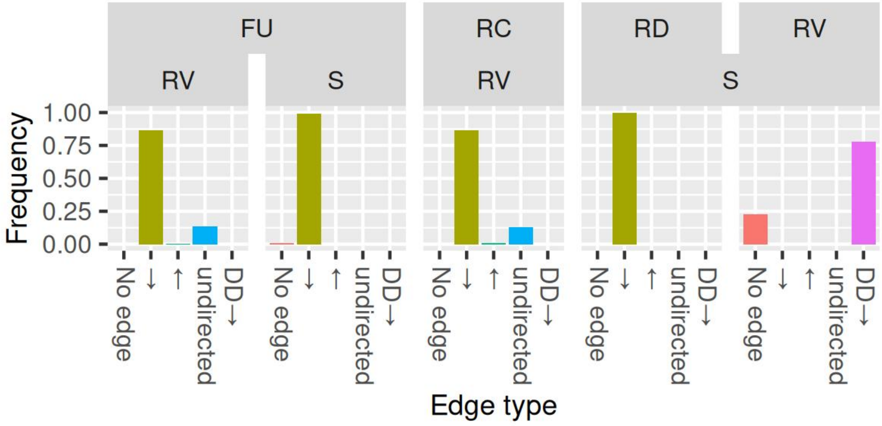

Figure S3. Discovered edge type frequencies.

The reported DAG was constructed from the edges with frequencies larger than 0.5. As shown in Figure S3, the discovered DAG results from edge frequencies larger than 0.75. This increases our confidence in the correctness of the discovered causal structure, which is crucial for the analysis of probabilistic actual causation.

Internally, we also conducted the causal discovery analysis with bootstrapping using Tetrad's GFCI, FGES, GRASP, and BOSS algorithm implementations (for all algorithms, we used the Degenerate Gaussian Likelihood Ratio Test (DG-LRT)). The edge frequencies slightly vary across algorithms, but the discovered structure is the same. For simplicity, we opted to report the PC results since it is a well-established algorithm with extensively studied properties (Malinsky and Danks, 2017; Glymour et al., 2019).

1.2 Assumption of no latent variables

The PC algorithm assumes no latent confounders or unobserved variables. In this respect, we rely on our analysis of the task and the data-generating process to support this assumption.

Based on the analysis of the task described in Section "4.2 DAG Variables" we assume that the variables used to represent the data-generating process of the simulation capture all the relevant causes of spillage. The variables represent the stochastic effect of 1) the containers' characteristics (capacities and diameters, expressed as the variables RC and RD) and 2) the poured amount (fullness and volume, expressed as the

variables FU and RV) on the probability of spilling (variable S). The randomness of the outcome S results from the interplay between RC, RD, FU, and RV and the behavior of the particles during the pouring movement. The parameters the physics engine uses (particle size and density) to simulate the particles' behavior have a constant value. Therefore, they are not included as DAG variables.

Additionally, the pouring movement was executed with constant rotation velocity and angle. Thus, these were not included as DAG variables. Based on these considerations, we are confident that we included all the relevant random variables of the data-generating process of the simulation and assume that there are no latent confounders or other unobserved variables with a causal effect on spillage.

2 IMPLEMENTATION OF THE NEURAL AUTOREGRESSIVE DENSITY ESTIMATORS

The implementation of the NADE networks is based on the source code provided by Garrido et al. (2021), publicly available in `https://github.com/Chechgm/causal_effect_estimation_using_nade` (access: 05.12.24). The following parameters were used:

- Neural network architecture: 2 hidden layers with 16 units each
- Activation function: hyperbolic tangent (Tanh)
- Optimizer: RMSProp
- Learning rate: 0.01

The neural networks were implemented using Pytorch (Paszke et al., 2019) (Version 1.10.0).

The hyper-parameters were selected based on the results reported by Garrido et al. (2021). In their extensive analysis of the performance of different hyper-parameters (hidden layers, number of units, and learning rates), they conclude that no single combination of hyper-parameters is superior to others. We selected two hidden layers with 16 units. We noticed that increasing the number of layers and units did not reduce the loss or improve the prediction performance of the network. Based on this empirical observation, which is in line with the observations of Garrido et al. (2021), we abstained from conducting any further systematic search or comparison of the hyper-parameter space.

3 EMPIRICAL SUPPORT TO THE CORRECTNESS OF THE CAUSAL MODEL

The analysis of probabilistic actual causation relies on the correctness of the causal structure and the estimated causal probabilities. It is important to note that the causal structure of the data-generating process is unknown. Therefore, no ground truth causal graph is available to benchmark the structure and the causal probabilities learned from the training dataset.

In simulation, we have control over the parameters used in each pouring trial. The actual movement of the particles during the pouring movement, which determines whether or not spillage occurs, depends on the interaction between the trial parameters and the physics simulation. This interaction between the trial parameters and the spillage outcome is unknown. Therefore, the benchmarking of the causal graph can only be conducted on the level of evaluating the spillage predictions against the ground truth. We evaluate the spillage predictions on a test dataset of 3000 pouring trials. The trial parameters of the test dataset were sampled from the same distribution used for the training dataset (see description in Section "4.2 DAG Variables"). For each trial, we compute the causal probability of spillage $P(S|do(FU, RC, RD, RV))$. If $P(S|do(FU, RC, RD, RV)) \geq 0.5$, the outcome prediction is labeled as $S = True$. We compare the predicted outcome with the actual outcome. The prediction results are summarized in the confusion matrix

shown in Figure S4. Among the spillage trials, the causal model predictions yield 90.8% true positives and 9.2% false negatives. On the other hand, the causal model predictions yield 94.5% true negatives and 5.5% false positives among the no-spillage trials. These results provide empirical support that the causal model corresponds with the ground truth data-generating process.

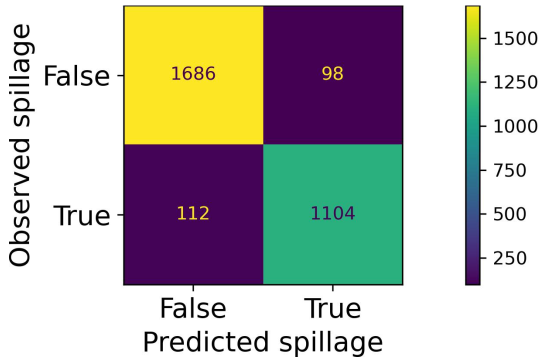

Figure S4. Confusion matrix.

REFERENCES

Garrido, S., Borysov, S., Rich, J., and Pereira, F. (2021). Estimating causal effects with the neural autoregressive density estimator. *Journal of Causal Inference* 9, 211–228. doi:10.1515/jci-2020-0007

Glymour, C., Zhang, K., and Spirtes, P. (2019). Review of causal discovery methods based on graphical models. *Frontiers in Genetics* 10. doi:10.3389/fgene.2019.00524

Malinsky, D. and Danks, D. (2017). Causal discovery algorithms: A practical guide. *Philosophy Compass* 13. doi:10.1111/phc3.12470

Paszke, A., Gross, S., Massa, F., Lerer, A., Bradbury, J., Chanan, G., et al. (2019). *PyTorch: an imperative style, high-performance deep learning library* (Red Hook, NY, USA: Curran Associates Inc.). 8026 – 8037
